# CC vs. CC-Plus: A Comparison between Two Cranial-to-Caudal Approaches for Laparoscopic Right Hemicolectomy: A Single-Center Retrospective Study

**DOI:** 10.3390/jpm14080781

**Published:** 2024-07-23

**Authors:** Yurong Jiao, Federico Maria Mongardini, Haiting Xie, Xinyi Zhou, Xiangxing Kong, Jihang Wen, Ludovico Docimo, Jun Li, Claudio Gambardella

**Affiliations:** 1 Department of Colorectal Surgery and Oncology (Key Laboratory of Cancer Prevention and Intervention, China National Ministry of Education, Key Laboratory of Molecular Biology in Medical Sciences, Zhejiang Province, China), The Second Affiliated Hospital, Zhejiang University School of Medicine, Hangzhou 310009, China; jiaoyurong@zju.edu.cn (Y.J.); xmuxhting@zju.edu.cn (H.X.); xinyizhou@zju.edu.cn (X.Z.); xiangxingkong@zju.edu.cn (X.K.); jihangwen@zju.edu.cn (J.W.); junli@zju.edu.cn (J.L.); 2 Division of General, Oncological, Mini-Invasive and Obesity Surgery, University of Study of Campania “Luigi Vanvitelli”, 80131 Naples, Italy; federicomaria.mongardini@unicampania.it (F.M.M.); ludovico.docimo73@gmail.com (L.D.)

**Keywords:** right hemicolectomy, minimal invasive hemicolectomy, cranio-to-caudal hemicolectomy, colorectal cancer

## Abstract

Background: Colorectal cancer is a leading cause of cancer-related deaths worldwide, with approximately 1.9 million new cases and over 935,000 deaths in 2020. Right-sided colon cancer, a subset of colorectal cancer, represents a significant health burden. Laparoscopic colon surgery has significantly improved postoperative recovery. The superiority of one approach or landmark over another is still argued about due to the lack of large-scale prospective studies. However, deep understanding both of the anatomical variation and characteristics of each approach is of extreme importance to minimizing adverse effects and maximizing patient benefit after laparoscopic right hemicolectomy. Among these, the cranial-to-caudal approach offers advantages such as reduced intraoperative blood loss, shorter operation time, and decreased risk of vascular injury. The purpose of this study is to compare the efficacy and safety of two cranial-to-caudal approaches for laparoscopic right hemicolectomy (LRH). Specifically, the study aims to evaluate the differences between the conventional cranial-to-caudal approach with medial ligation of the middle colic vein (MCV), and the cranial-to-caudal approach with cranial MCV ligation and surgical trunk sheath opening (CC-plus). The goal is to determine which method offers superior outcomes in terms of intraoperative blood loss, operation time, and overall patient recovery. Materials and Methods: This single-center retrospective study compared two cranial-to-caudal approaches for LRH. The study included 51 patients who underwent LRH between January 2021 and November 2023 at the Second Affiliated Hospital, Zhejiang University School of Medicine. Patients were divided into two groups: Group A (26 patients) used the cranial-to-caudal approach with medial ligation of the middle colic vein (MCV), and Group B (25 patients) used the cranial-to-caudal approach with cranial MCV ligation and surgical trunk sheath opening (CC-plus). General characteristics, intraoperative parameters, and postoperative outcomes were compared. Statistical analysis was performed using SPSS version 20.0, with significance set at *p* < 0.05. Results: There were no significant differences between the groups regarding age, gender, tumor location, or clinical staging. All patients achieved R0 resection with no perioperative deaths. The CC-plus group had significantly reduced intraoperative blood loss and shorter operation time compared to the CC group (*p* < 0.05). No significant differences were found in first postoperative exhausting time, first postoperative defecation time, and postoperative hospital stay between the two groups. Furthermore, no significant differences were evaluated in postoperative complications (surgical site infection (SSI), ileus or bowel obstruction, refractory diarrhea, anastomotic leakage, deep vein thrombosis (DVT), hemorrhage) between the two groups on a median follow up of 12.6 months. Pathological examination showed no significant differences in total lymph nodes dissected and tumor stage. Conclusions: The cranial-to-caudal approach with MCV ligation via the cranial approach (CC-plus) is a safe and effective method for LRH, offering advantages in terms of reduced operation time and intraoperative blood loss. This study’s findings suggest that the CC-plus approach may be superior to the conventional cranial-to-caudal approach.

## 1. Introduction

Colorectal cancer poses a significant threat to human health [1,2]. Laparoscopic right hemicolectomy (LRH) is a commonly used procedure for treating right-side colon cancer.

Laparoscopic right hemicolectomy (LRH) poses significant challenges because of the intricate blood supply to the right colon, the variability in individual anatomy, and the technical complexities involved in safely excising lymph nodes at the base of the middle colic artery while preventing injury to the duodenum and pancreas [3]. Various surgical approaches for LRH, such as medial-to-lateral, lateral-to-medial, caudal-to-cranial, and cranial-to-caudal (CC), have been documented in the literature. However, a definitive consensus on the optimal surgical method has not yet been established [4,5]. According to the Japanese Society for Cancer of the Colon and Rectum (JSCCR) guidelines, the cranial-to-caudal approach with complete mesocolic excision (CME) and D3 lymph node dissection is recommended for right hemicolectomy, aiming to improve surgical outcomes by ensuring thorough lymph node removal and minimizing vascular injury. These guidelines underscore the importance of detailed preoperative planning and precise anatomical knowledge to navigate the complexities of the right colon vasculature [6].

Recently, innovative laparoscopic techniques have been developed, including the pincer approach, the artery-first approach, and the uncinate process-first approach [7,8,9,10]. However, there is still a lack of data from randomized controlled trials to determine the best approach for LRH. The CC approach offers advantages such as reduced intraoperative blood loss, shorter operation time, and decreased risk of vascular injury [11]. For the CC approach LRH, there are two different ways according to the literature, depending on the management of the middle colic vein and surgical trunk [11]. Although the literature has reported that there are many vascular variations in the LRH, especially around the gastrointestinal trunk and its accessory vessels, the middle colic vein is consistently present during LRH [12]. Ligation of the superior right colic vein (SRCV) is performed at the confluence of the right gastroepiploic vein (RGEV), while management of the middle colic vein (MCV) is deferred to the middle approach in the literature by Yao Yang et al. [11]. Takeru Matsuda et al. divided the MCV at its root and present cranially approached radical lymph node dissection along the surgical trunk in a cranial-to-caudal manner during LRH. The authors consider this approach to be safe and useful [13]. The key difference between these two techniques was the timing and position for ligation of the MCV and management for the surgical trunk. However, considering the limitation of the Matsuda study design (single center, small sample size, and retrospective nature), it appeared hard to draw definitive conclusions on the advantages and disadvantages of these two approaches.

Hence, we carried out a retrospective study of a single center to compare the intra-, perioperative, and postoperative surgical outcomes of patients undergoing LRH with the conventional CC approach vs. patients treated with CC-plus at 12 months follow-up.

## 2. Patients and Methods

### 2.1. Study Design

This study was reported according to the STROBE statement for cohort studies [14] and was conducted according to the ethical principles stated in the Declaration of Helsinki. A retrospective monocentric study was conducted to assess the surgical outcomes of patients undergoing LRH with the conventional CC approach vs. patients treated with CC-plus at 12 months follow-up. Written informed consent was obtained from all patients.

### 2.2. Study Setting and Study Population

From January 2021 to November 2023, consecutive cases undergoing LRH at the department of Colorectal Surgery, Second Affiliated Hospital, Zhejiang University School of Medicine were included in the analysis. Inclusion criteria were: (1) confirmed resectable right-sided colon tumor (from cecum to proximal part of transverse colon) via colonoscope; (2) absence of significant contraindications for surgery; (3) ability to tolerate laparoscopic surgery.

The considered exclusion criteria were: (1) emergency surgery; (2) obstructive or perforated disease; (3) absence of right hemicolectomy during the operation for reasons other than conversion; (4) stage IV or T4b patients.

All patients received a routine preoperative clinical and instrumental diagnostic assessment with anamnestic data collection, accurate general and abdominal examination, a pancolonoscopy, laboratory blood tests, and cardiological and anesthetic evaluations. The oncological staging comprised a total body computed tomography (CT) scan with contrast medium. Each case was discussed, prior to surgery, at a weekly multidisciplinary meeting (MDT) of the colorectal unit, composed of oncologists, surgeons, radiologists, radiotherapists, and gastroenterologists.

After the referral for surgery, each patient received a detailed explanation of the procedure by the medical staff and had to sign a personalized informed consent form. Each procedure was performed by the same experienced surgical team (more than 300 laparoscopic colorectal operations per year).

Clinical data were retrospectively collected and included in this study.

To compare the intraoperative, perioperative, and postoperative data, patients were divided into two groups:

Group A: patients undergoing LRH with cranial-to-caudal approach with MCV ligation and surgical trunk sheath opened from the cranial way (top to bottom) (C-C plus group).

Group B: patients undergoing LRH with cranial-to-caudal approach and ligated the MCV from the medial approach (C-C group).

Clinical data were collected in an electronic database and retrospectively analyzed.

### 2.3. Surgical Procedure

**Figure 1:**
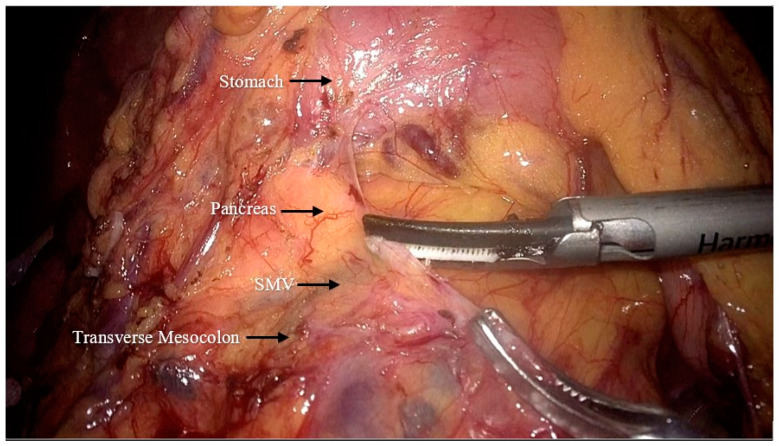
Incision of the transverse mesocolon at the lower margin of the pancreas to reveal the superior mesenteric vein (SMV: superior mesenteric vein).

**Figure 2:**
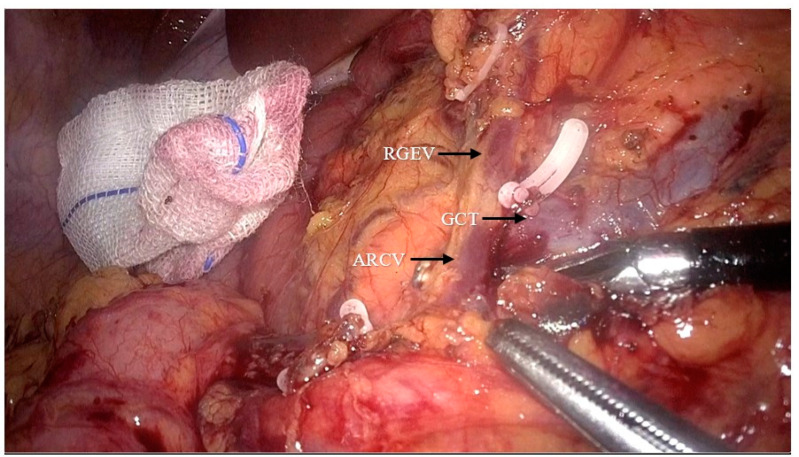
Dissection of the gastrocolic trunk and its branches to reveal and ligate the accessory right colic vein (ARCV: accessory right colic vein; RGEV: right gastroepiploic vein; GCT: gastrocolic trunk).

After completing preoperative preparation, the patient was placed in the reverse Trendelenburg position. The pneumoperitoneum pressure was set to 12 mmHg. A 10 mm trocar was inserted 2–3 cm below the umbilicus for the camera. Another 10 mm trocar was placed at the junction of the horizontal line (2 cm above the umbilicus) and the left anterior axillary line, with an additional auxiliary 5 mm trocar at the anti-McBurney point. The first assistant positioned a 5 mm trocar and a 10 mm trocar at specific points on the right side of the patient’s abdomen, while standing opposite to the surgeon who was located on their left side. After laparoscopic exploration of the abdominal cavity, the gastrocolic ligament was dissected and the transverse mesocolon was detached from the lower edge of the pancreas (Figure 1). The gastrocolic trunk and its associated vessels was located on the anterior surface of the pancreas, and the accessory right colic vein was ligated (Figure 2). The transverse and ascending colon were mobilized from the hepatic flexure. For patients from the C-C group, the cranial approach was finished by this step, while for patients from the C-C plus group, the cranial approach continued.

**Figure 3:**
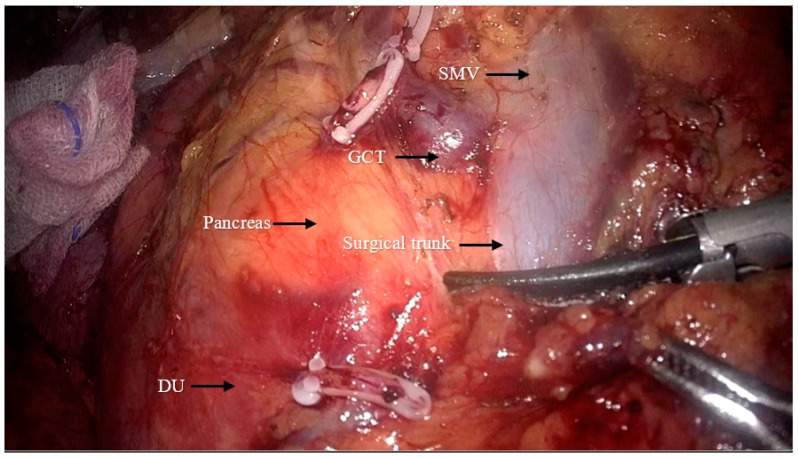
Exposition of the superior mesenteric vein (GCT: gastrocolic trunk; DU: duodenum; SMV: superior mesenteric vein).

**Figure 4:**
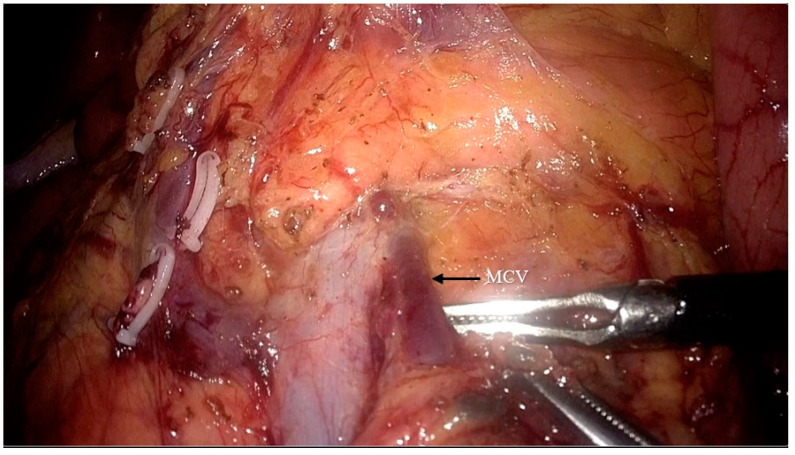
Dissection of the lymph nodes around the root of the middle colic vein (NO.223) and ligation of the middle colon vein (MCV: middle colic vein).

The superior mesenteric vein was exposed, and exposure of the surgical trunk continued by opening the sheath of the SMV (Figure 3). Lymph nodes around the root of the MCV (NO.223 from the Japanese Society for Cancer of the Colon and Rectum (JSCCR) guidelines [6]) were dissected and the MCV was ligated via the cranial approach (Figure 4).

Patient were subsequently placed in the Trendelenburg position and the small intestine was relocated to the upper abdomen. Along the caudal aspect of the ascending colon, following elevation of the mesenteric root of the small intestine, we meticulously dissected the “membranous bridge” between the small intestine mesentery, retroperitoneum, right mesocolon, and lateral peritoneum. Proceeding into the space delineated by Toldt’s fascia and advancing to the medial compartment, we completely liberated the ascending colon and its mesocolon. When we finished the dissection from the caudal approach by revealing the horizontal part of the duodenum and met the surgical field of the cranial approach, we turned the patient to reverse Trendelenburg position again.

**Figure 5:**
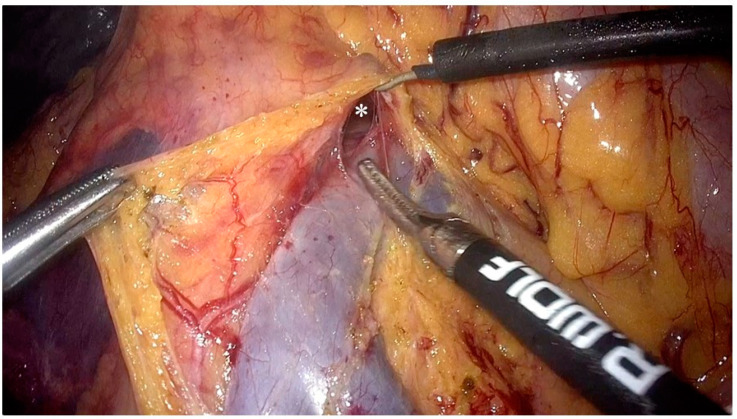
Dissection along the SMV and meeting the cranial area from the medial approach (*: the meeting point of the cranial approach and the caudal approach).

**Figure 6:**
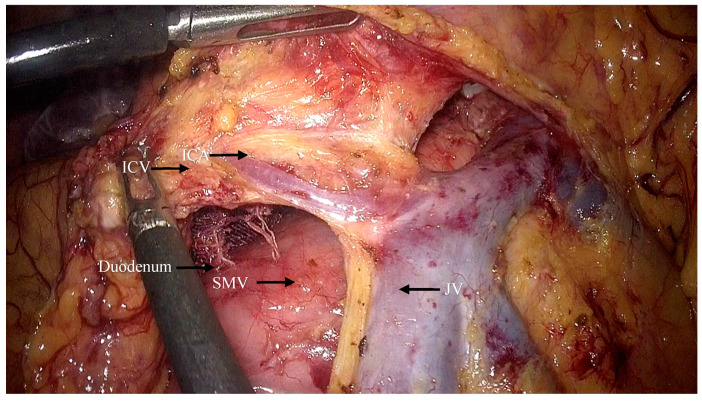
The ICA and ICV are revealed and later ligated (ICA: ileocolic artery; ICV: ileocolic vein; SMV: superior mesenteric vein; JV: jejunal vein).

**Figure 7:**
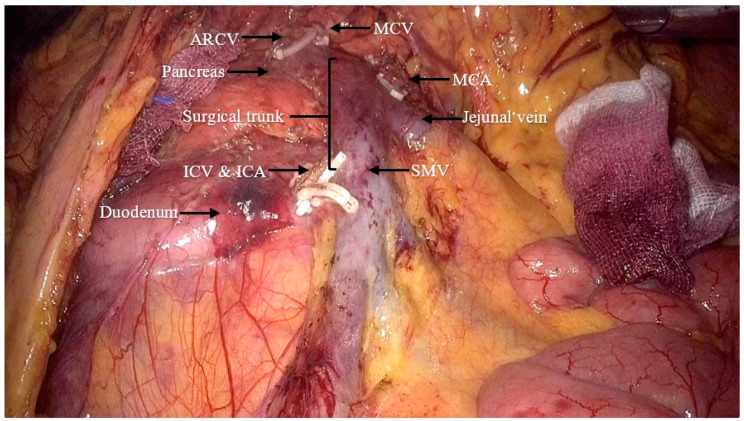
Surgical field for the LRH (ARCV: accessory right colic vein; MCV: middle colic vein; MCA: middle colic artery; ICV: ileocolic vein; ICA: ileocolic artery; SMV: superior mesenteric vein).

Dissection was conducted with a medial approach concerning the ileocolic artery and extended to the right margin of the superior mesenteric vein for the lymph node dissection area. We followed the dissection along the SMV until reaching the cranial region (Figure 5). The ileocolic artery, ileocolic vein, and middle colic artery were dissected (Figure 6). The right colon and part of ileum were resected. An end-to-side coloileal anastomosis was performed from a small laparotomy in the upper abdomen (Figure 7). The peritoneal cavity was washed and the bleeding foci were detected via laparoscopy. The residual bowel was arranged and the hepatorenal recess was drained.

### 2.4. Outcome Measures

Demographic preoperative characteristics, such as gender, body mass index, tumor location, and tumor clinical staging were recorded. Regarding the short-term outcome, the operation time, intraoperative blood loss, first postoperative exhausting time, first postoperative defecation time, postoperative hospital stay, harvested and positive lymph node number, and size of the tumor were recorded. The operation time was defined as the time from the initial insufflation to the end of the operation. The blood loss referred to the volume of blood after deducting the abdominal effusion before the abdominal cavity flushing plus the blood in the endoscopic gauze (nominally 10 mL per gauze).

During hospitalization, the following postoperative complications were analyzed: surgical site infection (SSI) [15], ileus or bowel obstruction, refractory diarrhea, anastomotic leakage, deep vein thrombosis (DVT), and hemorrhage. Moreover, the postoperative definitive pathology including the pathological classification, total number of lymph nodes dissected, and pathological staging was compared.

### 2.5. Study Outcomes

The primary outcome of the study focused on comparing the intraoperative and perioperative outcomes of patients undergoing LRH with the conventional CC approach vs. patients treated with CC-plus. Secondary outcomes included the assessment of postoperative complications and definitive pathology.

### 2.6. Statistical Analysis

SPSS version 20.0 was used for statistical analysis. The quantitative data were expressed as mean ± standard deviation ($\overset{-}{x}$ ± s) and compared with an independent sample *t*-test between the two groups. The qualitative data were expressed as frequency and percentage and compared with an χ^2^ test or Fisher’s exact test. A value of *p* < 0.05 was considered statistically significant.

## 3. Results

### 3.1. Study Population

Between January 2021 and November 2023, 329 patients were referred to our surgical department of right colon cancer and 79 received the CC approach during LRH. The inclusion criteria were satisfied by 51 subjects. Among these patients, 25 underwent the complete mesocolic excision via cranial-to-caudal (CC-plus) approach, while 26 underwent the conventional cranial-to-caudal (CC) approach. There were no significant differences in age, gender, tumor location, or clinical staging of the colon cancer between the two groups (*p* > 0.05) (Table 1).

All patients achieved R0 resection based on pathological examination of the surgical specimens. None required conversion to laparotomy, and no perioperative deaths were reported (Table 2). The medium postoperative follow-up was 12.6 ± 0.6 months (Range: 12.0–13.2), including regular surgical control in outpatient clinics.

### 3.2. Perioperative Outcomes

The study found significant differences in intraoperative blood loss and operation time between the two groups. The “CC-plus group” had significantly reduced intraoperative blood loss (30.00 mL vs. 50.00 mL, *p* = 0.001) and shorter operation time (168.24 min vs. 207.69 min, *p* = 0.004) compared to the “CC group”.

In terms of lymph node analysis, there were no significant differences between the two groups. Both groups had a similar number of harvested lymph nodes, with medians of 20 and 21 for the “CC-plus group” and the “CC group”, respectively. The median number of positive lymph nodes was also comparable, with 0.76 for the “CC-plus group” and 1.15 for the “CC group”.

The tumor sizes in both groups were comparable. The median length of the tumors was 4.28 cm in the “CC-plus group” and 4.18 cm in the “CC group”. Similarly, the median width of the tumors was 3.68 cm in the “CC-plus group” and 3.23 cm in the “CC group”, indicating no significant difference in tumor dimensions.

There were no significant differences in the incidence of first postoperative exhausting time, first postoperative defecation time, and postoperative hospital stay between the two groups (all *p* > 0.05) (Table 2).

Hospital recovery metrics, including the length of stay, time to first flatus, and time to first defecation, were similar between the two groups. Both groups had a median hospital stay of 7 days. The time to first flatus and first defecation did not differ significantly, suggesting that the recovery process was consistent across both groups (Table 3).

### 3.3. Postoperative Complication

Complication rates between the two groups were also comparable with a median follow up of 10.3 months (Table 4). The “CC-plus group” reported a total of three complications, while the “CC group” had five. Specific complications, such as surgical site infections, ileus or bowel obstruction, refractory diarrhea, anastomotic leakage, deep vein thrombosis, and hemorrhage, showed no significant differences in occurrence between the two groups. 

### 3.4. Postoperative Pathological Results

Negative proximal and distal resection margins were observed in all tumor specimens from both groups. There were no significant differences in the total number of lymph nodes dissected and tumor stage between the two groups (*p* > 0.05).

## 4. Discussion

Laparoscopic radical right hemicolectomy with complete mesocolic excision (CME) has become the standard approach for the radical resection of right colon cancer, facilitated by ongoing advancements in minimally invasive surgery and precise anatomical delineation [16]. The CME + D3 procedure, as recommended by the Japanese Colorectal Cancer Society (JSCCR) guidelines, is anticipated to yield favorable surgical outcomes [6,17]. However, the variability in the vascular anatomy of the right colon presents challenges in dissection and identification. Inadequate surgical techniques may result in significant vascular injury, thereby increasing surgical complexity and predisposing patients to severe postoperative complications [18,19]. Overall, the “CC-plus group” showed potential benefits in terms of shorter operation duration and reduced blood loss, while other postoperative outcomes, including recovery times and complication rates, remained similar to those in the “CC group”. This suggests that the “CC-plus group” may offer certain operative advantages without compromising patient recovery and safety. Yamaguchi et al. found that vascular variations are more common in the right colon, contributing to the prolonged learning curve of LRH. The management of the gastrocolic trunk is a significant challenge in LRH procedures and remains a focal point in current research [20]. We found that the branches of the gastrocolic trunk are more easily visualized and ligated using the cranial approach, particularly in patients with a high body mass index (BMI). The cranial-to-caudal approach aligns more closely with human embryonic development [21]. This technique involves accessing the avascular interstitial space from the head of the pancreas and the right gastroepiploic vessel. The dissection is then extended to the space behind the transverse colon, exposing the transverse colon, duodenum, and pancreatic head, allowing for the dissection of the gastrocolic trunk and middle colic vessels [22]. Cranial approach dissection more easily exposes the Henle trunk and its branches [9,21,22]. The middle colic vein can be identified from the posterior side of the transverse mesocolon, facilitating vascular ligation, and reducing bleeding events. Otherwise, bleeding in the Henle trunk area can be problematic due to the unseparated cranial region, which restricts space [11]. This limitation complicates hemostasis and may lead to severe bleeding and conversion to open surgery due to the restricted visibility associated with the midline approach. The cranial-to-caudal approach with medial ligation of the middle colic vein (CC-plus) for laparoscopic right hemicolectomy demonstrated significant advantages over the conventional cranial-to-caudal approach. These included reduced intraoperative blood loss and shorter operation time, suggesting that the CC-plus approach may be superior in terms of intraoperative efficiency and patient safety. This analysis highlights the importance of the CC-plus approach in improving surgical outcomes for patients undergoing LRH, though the study acknowledges the need for larger, prospective studies to confirm these findings. Following the ligation of the middle colic vein (MCV) and dissection of the surgical trunk via the cranial approach, transitioning to the midline approach from below upwards becomes significantly faster [11,21]. After transecting the ileocolic vessels, dissection along the superior mesenteric vein (SMV) typically converges with the cranial dissection area, thus accelerating the surgical process.

The current study has several limitations to address: its retrospective design, the single-center experience, the limited sample size, and lack of long-term follow-up. There is a need for prospective randomized controlled studies with larger sample sizes and longer follow-up periods to comprehensively investigate the impacts of various surgical interventions on LRH. Moreover, there is a statistical difference between the tumor location in the groups, with a greater incidence of hepatic flexure lesions in the CC Group. The hepatic flexure tumor is more challenging to remove and has a high frequency of lymph node metastasis in the middle colic vein area, determining an impact on the surgical outcomes.

## 5. Conclusions

The cranial-to-caudal approach with MCV ligation via the cranial approach (CC-plus) is a safe and effective method for LRH, offering advantages in terms of reduced operation time and intraoperative blood loss. This study’s findings suggested that the “CC-plus group” had advantages in terms of operation time and blood loss. The cranial-to-caudal approach with ligation of the MCV via cranial approach is a safe and effective method for LRH operations. Additional, larger, prospective comparative studies are needed to address this issue.

## Figures and Tables

**Table 1:** Demographic and clinicopathological features (CC-Plus vs. CC).

		Group A CC-Plus 25 Patients	Group B CC 26 Patients	*p* Value
Gender	M	11 (44%)	18 (69.2%)	0.069
	F	14 (56%)	8 (30.7%)	
Age		61.7 ± 9.1	63.7 ± 12.9	0.526
BMI		22.7 ± 3.3	22.9 ± 2.5	0.833
T stage	0	1 (4%)	0	0.353
	1	2 (8%)	3 (11.6%)	
	2	4 (16%)	1 (3.8%)	
	3	15 (60%)	15 (57.7%)	
	4	3 (12%)	7 (26.9%)	
N stage	0	17 (68%)	14 (53.8%)	0.553
	1	6 (24%)	8 (30.8%)	
	2	2 (8%)	4 (15.4%)	
AJCC stage	0	1 (4%)	0	0.462
	1	5 (20%)	3 (11.6%)	
	2	11 (44%)	10 (38.4%)	
	3	8 (32%)	13 (50%)	
Tumor location	Ascending colon	13 (52%)	3 (11.6%)	<0.001
	Hepatic flexure	9 (36%)	20 (76.9%)	
	Transverse colon	3 (12%)	0	
	cecum	0	3 (11.6%)	

CC Plus: cranial-to-caudal approach with MCV ligation via the cranial approach. CC: conventional cranial-to-caudal approach.

**Table 2:** Quality comparison in laparoscopic right hemicolectomy between two surgical methods.

	Group A CC-Plus 25 Patients	Group B CC 26 Patients	*p* Value
Operation duration (minutes) ^x^	168.2 ± 28.9	207.7 ± 57.8	0.004
Estimated blood loss (mL) *	30.0 (20.0–50.0)	50.0 (45.0–50.0)	0.001
Harvested LN number (n) *	20 (16–29)	21 (16–28)	0.962
Positive LN number (n) *	0.76 (0–5)	1.15 (0–6)	0.369
Tumor length (cm) *	4.3 (1.7–8.0)	4.18 (1.8–8.5)	0.867
Tumor width (cm) *	3.7 (0.7–5.5)	3.23 (1.6–7.5)	0.687

* mean value ± range; ^x^ mean value ± standard deviation; LN: lymph nodes.

**Table 3:** Comparison in perioperative outcomes of right-sided colon cancer.

	Group A CC-Plus 25 Patients	Group B CC 26 Patients	*p* Value
LOS (day) *	7.0 (6.5–9.0)	7.0 (6.0–8.0)	0.969
Flatus passage (day) *	3.0 (2.0–3.0)	3.0 (2.0–4.0)	0.203
Defecation passage (day) *	4.0 (4.0–6.0)	4.0 (4.0–5.2)	0.852

* mean value ± Range, LOS: length of stay.

**Table 4:** Perioperative Complication with Clavien–Dindo classification on median follow up of 12.6 ± 0.6 months.

	Group A CC-Plus 25 Patients	Group B CC 26 Patients	*p* Value
Complications (n)	3 (12%)	5 (19.2%)	0.477
Clavien–Dindo Grade I-II	2 (8%)	4 (15.4%)	0.413
Clavien–Dindo Grade III-IV	1 (4%)	1 (3.8%)	0.977
SSI	2 (8%)	1 (3.8%)	0.528
Ileus or Bowel Obstruction	1 (4%)	2 (7.7)	0.575
Refractory diarrhea	0	0	-
Anastomotic leakage	0	1 (3.8%)	0.322
DVT	0	0	-
Hemorrhage	0	1 (3.8%)	0.322
Mortality	0	0	-

SSI: surgical site infection; DVT: deep vein thrombosis.

## Data Availability

The datasets used and/or analysed during the current study are available from the corresponding author on reasonable request.
